# Supplementary material for: Haruspex: A Neural Network for the Automatic Identification of Oligonucleotides and Protein Secondary Structure in Cryo‐Electron Microscopy Maps
Source: Angew Chem Int Ed Engl. 2020 May 11;59(35):14788–95. doi: 10.1002/anie.202000421 (PMC7497202; doi:10.1002/anie.202000421)
Supplement: Supplementary file 1 — Supplementary [file ANIE-59-14788-s001.pdf]

Angewandte Chemie
Eine Zeitschrift der Gesellschaft Deutscher Chemiker

Supporting Information

Haruspex: A Neural Network for the Automatic Identification of Oligonucleotides and Protein Secondary Structure in Cryo-Electron Microscopy Maps**

*Philipp Mostosi, Hermann Schindelin, Philip Kollmannsberger, and Andrea Thorn**

anie_202000421_sm_miscellaneous_information.pdf

Supporting Information: Computational Methods

Training Data

We queried the Electron Microscopy Data Bank (EMDB) for all single particle Cryo-EM maps with a resolution ≤4 Å, for which corresponding protein models were available in the Protein Data Bank in Europe (PDBe), yielding 576 map and model pairs as of February 2018. We filtered these EMDB/PDB pairs by the following three criteria: (1) Convincing visual fit between map and model; (2) presence of at least one annotated α-helix or β-sheet; and (3) preference of the highest resolution map in case the same authors deposited several instances of the same macromolecular complex. Maps with severe misfits, misalignments, or models without corresponding reconstruction densities, and vice versa, were discarded. After applying these criteria, we retained 293 map/model pairs for generating the training data (see Table 1, below).

To extract secondary structure information from the PDB data, we developed a custom parser for the PDBML[1] format based on xmltodict. To obtain additional secondary structure information, we implemented a variant of the DSSP algorithm[2] without strand direction, and a torsion angle based secondary structure detection inspired by STRIDE[3]: annotated or DSSP-detected secondary structures were extended by neighboring amino acids if they matched the same Ramachandran profile.

Annotation of reconstruction maps

For every entry pair, the augmented model was then superimposed on the map and all voxels within 3 Å of a protein backbone atom, or, in the case of nucleotides, within 3 Å of any non-Hydrogen atom, were assigned to the respective class (helix, sheet or nucleotide) if their value was higher than ½ of the average backbone density of the helix, sheet or nucleotide in question. Secondary structures with a backbone standard deviation of <2 σ and atoms without secondary structure assignment were either excluded, as they were likely incorrectly modelled, misfitted, or flexible structures, or labelled as 'unassigned'. For some training data pairs, such as virus capsids, only small or partial protein models were deposited for large Cryo-EM maps, resulting in well-defined high-density regions without model coverage. These regions would not get annotated and hence result in false positives if the network tried to predict the actual structure. To mitigate this, all voxels with density ≥ 1.0 r.m.s.d. but not within 5 Å of a model atom with density ≥ 1.0 r.m.s.d. were masked as unmodeled density and hence did not contribute to training.

Since our network generated a single class label as output, the reconstruction density of the secondary structures must be converted to a strict assignment to one of the three classes in order to be used as training examples. For each secondary structure, the reconstruction map density was multiplied by the backbone standard deviation and rescaled to an output density between zero and one (corresponding to 0.5 and 1.0 times the average backbone density of the local secondary structure element) for each label type. The highest channel value determined the voxel class. If multiple channels shared the same value, sheets took precedence over oligonucleotides, which took precedence over helices. Voxels where all channel values were below 0.01 were assigned the 'unassigned' class. Finally, reconstruction maps were rescaled to a voxel size of 1.1 Å if they were outside of [1.0; 1.2] Å.

Generation of training segments

To generate the 70^3 voxel sized segments needed for training, candidate volumes were sampled from the entire map, and segments with a mean backbone density < 3.0 r.m.s.d., less than 5% annotated volume, or less than 100 atoms with standard deviation ≥ 1.0 r.m.s.d. were discarded. This resulted in altogether 2183 training segments, of which 110 segments (5%) were held back for evaluation during training. To generate additional segments for training, we applied rotations in steps of 90° around all three axes, resulting in 24 rotated versions of each segment that could all be used as separate training volumes since the convolutional network is not rotation-invariant. Segments were further augmented during training by using a randomly translated 40^3 sub-cube for each step.

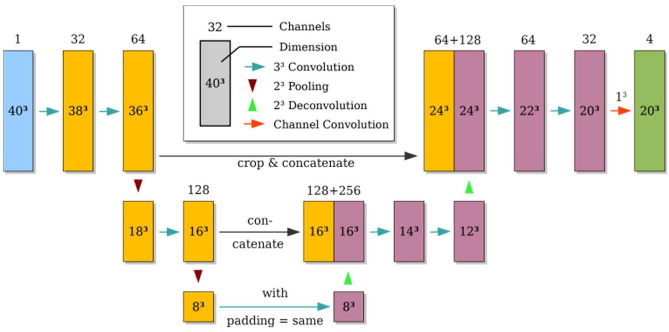

Figure 4. Haruspex neural network architecture. The network consists of multiple interconnected layers, shown as rectangular boxes. The layers are connected by convolution and pooling operations (arrows). Layer height represents the level of abstraction: lower layer data, generated by pooling operations, contain more abstract representations of the map. Input data (blue) is fed into the downconvolutional arm (yellow) in order to extract valuable information, which is then combined with previously discarded information through concatenations in the upconvolutional arm (purple) to compute annotated output data (green) for a subsection (20^3) of the input volume (40^3). Our network consists of two encoder blocks, containing altogether three convolutional layers (3x3x3) and two pooling layers. This is followed by two decoder blocks, one with upconvolution followed by two 3x3x3 convolutions and 128 feature channels, and one with upconvolution followed by two 3x3x3 convolutions with 64 and 32 feature channels, with concatenated sections of the corresponding layer in the encoding part. The output part consists of a final 1x1x1 convolution followed by a soft-max output layer. This results in 13 layers in total (12 + 1 convolution at bottom).

Network Architecture

We used a state-of-the-art U-Net-like encoder-decoder architecture[4,5] (see Fig. 4) with a single input channel (the reconstruction density). This architecture is a variant of so-called fully convolutional networks where spatial information and object details are encoded, reduced by pooling layers and then recovered again with up-sampling or transpose convolutions; the term U-Net arises from the U-like shape of the data flow. The encoding branch consisted of two 3x3x3 convolutional layers with 32 and 64 feature channels, respectively, followed by max-pooling layers. Another convolutional layer with 128 feature channels followed by a max-pooling layer finally resulted in an 8^3 cube with 128 feature channels at the deepest layer of the network. This cube was passed through another convolutional layer with the same data padding in order to preserve its dimensions. A fully connected layer was considered, but not

chosen due to its high memory and performance cost. The decoding branch of the U-Net was made of two blocks, each consisting of a deconvolution followed by two 3x3x3 convolutions (128 feature channels in the first, 64 and 32 channels in the second block to restore symmetry) with concatenated sections of the corresponding layer in the encoding part. The output part consists of a final 1x1x1 convolution followed by a soft-max output layer. The output layer reproduced the central 20^3 voxel cube of the input layer in four annotation channels representing co-dependent probabilities for the four classes (helix, sheet, nucleotide, unassigned) summing up to one. The highest channel value determined the predicted class. Implementation was realized using TensorFlow[6]. The network was trained end-to-end by comparing the predicted class of each voxel to the annotated EMDB model using cross-entropy loss, back propagating the error through the network, and adapting the network weights to iteratively minimize the error.

Network Training

The network was trained for 40,000 steps on training batches of 100 random segment pairs per step corresponding to 80 epochs, using ADAM stochastic optimization[7] with a learning rate of 0.001, β_1 = 0.9, β_2 = 0.999 and ε = 0.1. Error assignment for backpropagation was performed using cross-entropy loss, where the target class was represented in one-hot encoded binary format (1 for the target class, 0 for the other three classes). To account for class imbalance, voxels were weighted according to overall class occurrence in the training data. Furthermore, non-true negatives were weighted 16-fold stronger than true negatives.

[1] J. Westbrook, N. Ito, H. Nakamura, K. Henrick, H. M. Berman, *Bioinformatics* **2004**, *21*, 988–992.

[2] W. Kabsch, C. Sander, *Biopolymers: Original Research on Biomolecules* **1983**, *22*, 2577–2637.

[3] D. E. Tronrud, D. S. Berkholz, P. A. Karplus, *Acta Crystallographica Section D Biological Crystallography* **2010**, *66*, 834–842.

[4] Ö. Çiçek, A. Abdulkadir, S. S. Lienkamp, T. Brox, O. Ronneberger, in *International Conference on Medical Image Computing and Computer-Assisted Intervention*, Springer, **2016**, pp. 424–432.

[5] T. Falk, D. Mai, R. Bensch, Ö. Çiçek, A. Abdulkadir, Y. Marrakchi, A. Böhm, J. Deubner, Z. Jäckel, K. Seiwald, et al., *Nature Methods* **2019**, *16*.

[6] M. Abadi, A. Agarwal, P. Barham, E. Brevdo, Z. Chen, C. Citro, G. Corrado, A. Davis, J. Dean, M. Devin, et al., *TensorFlow: Large-Scale Machine Learning on Heterogeneous Distributed Systems*, **2015**.

[7] D. P. Kingma, J. Ba, *arXiv preprint arXiv:1412.6980* **2014**.

Table 1. List of EMDB/PDB entries used as training data.

EMDB	PDB	EMDB	PDB	EMDB	PDB	EMDB	PDB	EMDB	PDB	EMDB	PDB
2278	3j2v	4140	5m1j	8123	5it7	3570	5mup	6675	5wq7	8605	5us9
2513	4ci0	4146	5m32	8138	5iyd	3571	5muu	6676	5wq8	8606	5uu5
2566	3j6b	4147	5m3f	8139	5iz7	3574	5mv5	6687	5wtf	8608	5uvn
2650	3j7q	4148	5m3m	8175	5jte	3583	5mz6	6698	5x0m	8615	5uyk
2762	3j7y	5199	4v7q	8176	5ju8	3593	5n61	6699	5x0x	8616	5uyl
2764	3j80	5256	3izx	8184	5jzg	3601	5n8o	6703	5x58	8617	5uym
2773	4uy8	5495	3j26	8185	5jzh	3618	5np6	6705	5x5b	8618	5uyn
2787	4v19	5499	3j2p	8189	5k0u	3624	5nd8	6709	5x8p	8619	5uyp
2807	3j8h	5623	3j9i	8194	5k12	3630	5ned	6710	5x8r	8620	5uyq
2832	3j92	5764	3j4u	8237	5kcr	3654	5nj3	6711	5x8t	8622	5uz5
2847	5afi	5776	3j5q	8238	5kcs	3656	5njt	6732	5xlr	8641	5v7q
2857	5adx	5926	3j6q	8253	5kip	3695	5nsr	6733	5xmi	8642	5v7v
2876	3j9m	5995	3j7h	8279	5kps	3713	5nwy	6742	5xnm	8645	5v93
2913	5aj3	6000	3j7l	8281	5kpw	3730	5o2r	6743	5xnn	8650	5va1
2924	4ui9	6037	3j7x	8282	5kpx	3747	5o5b	6744	5xno	8658	5vc7
2938	4ug0	6057	3j7z	8289	5kuf	3748	5o5j	6752	5xs5	8697	5vjh
2981	5a0q	6224	3j9c	8314	5l35	3750	5o60	6757	5xs7	8708	5vly
2984	5a1a	6239	3j9d	8315	5sy1	3751	5o61	6770	5xsy	8712	5vms
3013	5a32	6240	3j9e	8331	5szs	3771	5oac	6771	5xtb	8713	5vn3
3037	3jaj	6306	3j9w	8333	5t0c	3817	5oik	6772	5xtc	8732	5vt0
3045	3jan	6311	3j9y	8342	5t15	3824	5ojs	6773	5xtd	8746	5vya
3047	3jam	6324	3ja7	8343	5t2a	3842	5ool	6774	5xte	8762	5w3m
3061	5a63	6337	5a22	8345	5t2c	3847	5oql	6775	5xth	8764	5w3s
3129	5ac9	6338	3ja8	8354	5t4d	3908	6eoj	6777	5xwy	8778	5w68
3130	5aca	6371	3jaz	8361	5t5h	4014	5l8q	6778	5xxb	8782	5w81
3151	5apo	6374	3jb0	8369	5t7v	4015	5l93	6780	5xxu	8784	5w9i
3152	5apn	6394	3jb4	8372	5t9m	4038	5ld2	6784	5xy3	8795	5wc3
3178	5fj8	6398	3jb5	8373	5t9n	4050	5li0	6788	5xyi	8827	5wfe
3218	5flm	6408	3jb6	8397	5tc1	4053	5lii	6789	5xym	8840	5wj5
3231	5fmg	6413	3jb9	8399	5tcq	4055	5lj3	6790	5xyu	8859	5wlc
3239	5fn4	6435	3jbg	8402	5tcu	4063	5lk7	6829	5yhq	8860	5wln
3245	3jc2	6455	5an8	8409	5tj5	4068	5lki	7019	6ayf	8881	5wpq
3246	5foj	6478	3jbs	8435	5tqq	4070	5lks	7030	6b0x	8882	5wpt
3295	5ftj	6480	3jbt	8454	5tr1	4071	5ll6	7036	6b2z	8883	5wpv
3331	5g2x	6486	3jbv	8461	5uar	4073	5lmn	7051	6b47	9400	5bk4
3337	5fwk	6487	3jbx	8477	5u07	4080	5lmu	7059	6b6h	9512	5gjr
3388	5g05	6488	3jby	8478	5u0a	4093	5lnk	7071	6b9q	9515	5gjw
3434	5m3l	6526	3jcl	8481	5u1c	4115	5lwi	7073	6baa	9517	5gka
3446	5m5w	6534	3jc6	8482	5u1d	4121	5lza	8003	5gag	9518	5gky
3460	5mbv	6551	3jcf	8506	5u4j	4124	5lzd	8011	5gam	9519	5gkz
3461	5mc6	6555	3jci	8511	5u6o	4125	5lze	8014	5gap	9524	5gm6
3490	5mdw	6559	3jcj	8512	5u6p	4130	5lzs	8015	5gaq	9539	5gup
3508	5mgp	6583	3jcs	8515	5u70	4131	5lzt	8064	5hx2	9564	5h0r
3525	5mlc	6584	5imq	8521	5u9f	4132	5lzu	8072	5i68	9565	5h0s
3531	5mmi	6615	3jct	8522	5u9g	4133	5lzv	8099	5ipi	9569	5h4p
3532	5mmj	6617	3jcu	8540	5udb	4134	5lzw	8107	5iqr	9570	5h1q
3533	5mmm	6635	3jd2	8576	5umd	4135	5lzx	8117	5irx	9572	5h1s
3539	5mps	6656	5h3o	8598	5urf	4136	5lzy	8118	5irz	9575	5h37
3551	5mrc	6667	5h5u	8604	5us7	4137	5lzz	8119	5is0		

Table 2. List of EMDB/PDB entries used as test data with individual test results.

EMDB	PDB	True Positives (%)	False Positives (%)	False Negatives (%)	Recall[a] (%)	Precision[a] (%)	Remarks
0001	6gh5	64.8	31.2	4.0	94.2	67.5	Fig. 3F
0011	6gjc	75.7	20.4	4.0	95.0	78.8	
0065	6gtg	60.6	20.0	19.3	75.8	75.2	
0088	6gy6	91.5	6.7	1.7	98.1	93.2	
0089	6gyb	66.2	29.7	4.1	94.1	69.0	
0097	6gyu	78.7	18.4	2.8	96.5	81.0	
0128	6h25	70.7	24.1	5.2	93.1	74.6	
0133	6h3i	48.4	49.7	1.9	96.3	49.3	Fig. 3B
0136	6h3n	66.8	29.9	3.3	95.3	69.1	
0150	6h6f	78.4	17.9	3.7	95.5	81.4	
0180	6hbc	73.8	18.2	8.0	90.2	80.3	
0199	6hcy	84.8	15.0	0.2	99.7	85.0	
0227	6hiq	77.8	18.0	4.2	94.9	81.2	
0257	6hra	78.4	20.1	1.5	98.1	79.6	
0279	6huj	78.7	18.1	3.2	96.1	81.3	
0281	6hum	78.5	18.2	3.3	96.0	81.1	
0289	6hwh	72.3	22.9	4.7	93.9	75.9	
0308	6hyd	80.3	15.5	4.1	95.1	83.8	
0336	6n3q	82.1	16.1	1.8	97.9	83.6	
0339	6n4b	71.2	27.0	1.8	97.5	72.5	
0341	6n4q	71.8	13.1	15.1	82.6	84.6	
0345	6n51	69.5	27.5	3.0	95.9	71.7	Fig. 3E
3984	6ez8	74.0	18.9	7.1	91.3	79.7	
4219	6fay	50.7	45.9	3.4	93.6	52.5	
4230	6fbv	69.2	19.8	10.9	86.4	77.7	
4264	6fhs	74.7	21.8	3.6	95.5	77.4	
4281	6fn1	75.3	18.8	6.0	92.7	80.0	
4287	6fo1	84.8	13.8	1.4	98.4	86.0	
4297	6fq5	92.7	5.9	1.4	98.5	94.0	Fig. 3A
4302	6ft6	75.4	20.5	4.0	95.0	78.6	
4339	6g1k	85.9	13.6	0.5	99.4	86.3	
4345	6g2j	75.3	21.5	3.2	96.0	77.8	
4358	6g79	70.1	16.8	13.1	84.3	80.6	
4362	6g8z	88.3	7.9	3.8	95.8	91.8	
4386	6gct	84.0	15.8	0.2	99.8	84.2	
6822	5yd1	88.4	10.8	0.9	99.0	89.1	
6856	5yx9	86.7	12.5	0.8	99.1	87.4	
6877	5z1w	75.1	22.2	2.7	96.6	77.1	
6901	5z96	86.4	11.5	2.1	97.6	88.3	
6917	5zdh	75.2	17.6	7.2	91.2	81.1	
6929	5zgb	75.9	18.7	5.4	93.3	80.2	
6941	5zr1	82.5	11.9	5.7	93.6	87.4	
6991	6a70	70.3	27.0	2.7	96.3	72.2	
6997	6a95	82.8	11.6	5.6	93.6	87.7	
6998	6a96	66.3	27.9	5.8	91.9	70.4	
7075	6bbj	83.1	12.4	4.5	94.9	87.0	
7299	6bwi	74.9	23.1	2.1	97.3	76.4	
7320	6c04	70.6	27.1	2.3	96.8	72.3	
7348	6c6l	87.9	10.5	1.6	98.2	89.3	
7352	6c70	87.7	11.6	0.7	99.2	88.3	

[a] As defined in the main article:
recall = true positives / (true positives + false negatives)
precision = true positives / (true positives + false negatives)

EMDB	PDB	True Positives (%)	False Positives (%)	False Negatives (%)	Recall[a] (%)	Precision[a] (%)	Remarks
7435	6c9a	87.7	8.4	3.9	95.8	91.2	
7442	6caj	61.1	9.2	29.8	67.2	86.9	
7460	6cdi	56.8	41.3	1.9	96.8	57.9	
7464	6ces	65.9	30.3	3.7	94.6	68.5	
7468	6cfw	79.5	18.9	1.6	98.1	80.8	
7482	6cjq	87.3	12.0	0.7	99.2	87.9	
7516	6cm3	61.1	36.2	2.7	95.8	62.8	
7526	6cmx	56.3	37.8	6.0	90.4	59.8	
7535	6cnj	69.8	23.0	7.2	90.7	75.2	
7537	6cnm	91.0	5.7	3.4	96.4	94.1	
7542	6co7	86.5	10.6	2.9	96.8	89.1	
7544	6coy	92.1	4.2	3.7	96.1	95.7	
7573	6crv	74.3	14.5	11.2	86.9	83.7	
7609	6csx	83.4	11.9	4.7	94.6	87.5	
7620	6cud	88.5	7.2	4.4	95.3	92.5	
7637	6cv9	79.4	20.6	0.0	100.0	79.4	
7783	6d03	72.5	26.6	0.9	98.8	73.2	
7808	6d6q	70.8	24.1	5.2	93.2	74.6	
7823	6d7l	76.1	23.2	0.7	99.1	76.7	
7826	6d7w	75.0	21.5	3.5	95.5	77.8	
7835	6d9h	80.9	16.1	3.0	96.4	83.4	
7844	6dbj	77.2	17.7	5.2	93.7	81.4	
7868	6dde	67.2	12.2	20.6	76.6	84.7	
7882	6dg7	79.3	19.5	1.2	98.5	80.3	
7942	6dju	70.4	25.2	4.4	94.1	73.6	
7959	6dlz	75.3	22.5	2.3	97.1	77.0	
7965	6dmr	82.6	15.4	2.0	97.6	84.3	
7968	6dmy	75.1	22.4	2.5	96.8	77.0	
7971	6dnf	82.3	10.6	7.1	92.1	88.6	
7981	6dqn	73.9	5.8	20.3	78.5	92.7	
7999	6drj	76.8	13.3	9.9	88.6	85.2	
8909	6ds5	64.7	32.0	3.3	95.1	66.9	
8911	6dt0	85.3	14.2	0.6	99.3	85.8	
8912	6du8	61.3	21.5	17.2	78.1	74.0	
8922	6dw0	66.0	21.2	12.8	83.7	75.7	
8953	6e14	55.9	33.2	10.9	83.7	62.7	
8957	6e1m	82.3	9.1	8.6	90.5	90.1	
8959	6e1o	82.2	17.8	0.0	100.0	82.2	
8962	6e2g	75.7	23.4	0.9	98.8	76.3	
8969	6e2r	62.6	26.9	10.5	85.7	69.9	
8978	6e3y	72.8	20.1	7.1	91.1	78.4	
9000	6e7p	79.7	12.7	7.6	91.3	86.3	
9012	6e9d	46.8	44.2	9.0	83.9	51.5	
9013	6e9e	81.3	16.6	2.0	97.6	83.0	
9024	6ebk	80.5	14.5	5.0	94.2	84.7	
9032	6edo	69.9	29.3	0.8	98.8	70.5	
9065	6mb3	61.7	31.9	6.3	90.7	65.9	
9066	6mcb	72.9	20.0	7.1	91.1	78.5	
9103	6mdp	72.4	26.6	1.0	98.6	73.1	
9104	6mdr	75.9	19.2	4.9	93.9	79.8	
9112	6mgv	85.4	6.1	8.5	91.0	93.4	
9116	6mhq	91.4	7.6	1.0	98.9	92.3	

EMDB	PDB	True Positives (%)	False Positives (%)	False Negatives (%)	Recall[a] (%)	Precision[a] (%)	Remarks
9117	6mhs	86.0	11.6	2.4	97.3	88.1	
9230	6msm	84.9	10.8	4.3	95.2	88.8	
9244	6mu2	66.4	16.9	16.7	79.9	79.8	
9256	6muu	73.9	20.6	5.5	93.0	78.2	
9277	6mwq	60.5	22.3	17.2	77.9	73.1	
9318	6n1r	74.5	19.6	5.9	92.7	79.2	
9326	6n28	82.6	7.3	10.1	89.1	91.9	
9382	6niy	73.6	21.0	5.4	93.2	77.8	
9588	6acc	61.1	26.2	12.7	82.9	70.0	
9590	6acf	78.2	13.0	8.8	89.8	85.7	Fig. 3C
9616	6agb	76.0	13.5	10.5	87.9	84.9	
9617	6agf	85.5	13.1	1.4	98.4	86.8	
9627	6ahu	70.5	18.8	10.7	86.8	79.0	Figs.1,2B,2C,2D
9648	6idf	81.1	13.8	5.1	94.1	85.4	
9657	6ifu	70.6	24.3	5.2	93.1	74.4	
9682	6ijz	82.3	16.1	1.6	98.1	83.7	
9708	6iqw	66.2	20.1	13.7	82.8	76.7	
9747	6ixh	68.8	28.3	3.0	95.9	70.9	Fig. 3D
9751	6iyc	81.7	15.8	2.5	97.0	83.8	

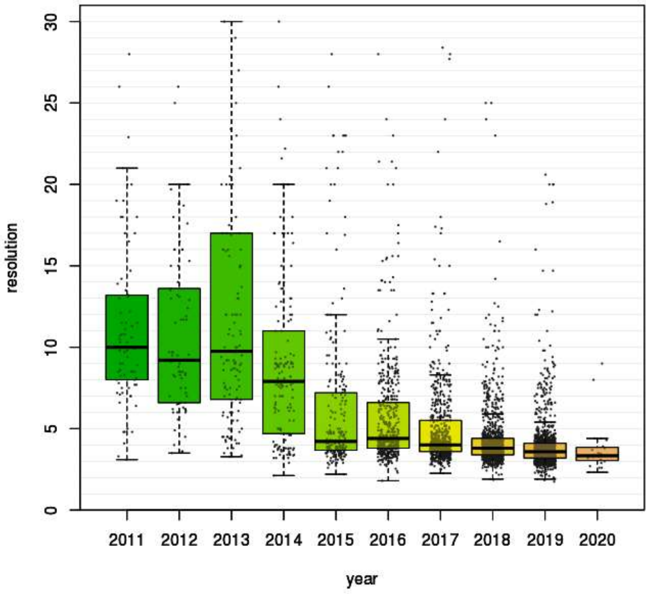

Figure 5. Resolution of depositions vs. year. These boxplots show the trend of annual average resolution for published EM maps/structures in the Electron Microscopy Data Bank (EMDB). We used the main resolution as given in the deposition for entries deposited between 1/1/2011 and 3/3/2020. Entries without resolution were omitted. The midline of the boxes corresponds to the median values, which are 3.8 Å for 2018, 3.6 Å for 2019 and 3.3 Å for 2020.
